# 537. First Week Post-Operative C-Reactive Protein Kinetics Show Different Patterns of Association with Infection Depending on the Type of Surgery

**DOI:** 10.1093/ofid/ofac492.590

**Published:** 2022-12-15

**Authors:** Anna M van Boekel, Siri L van der meijden, Bart F Geerts, Sesmu M Arbous, Mark G de Boer

**Affiliations:** Leiden University Medical Center, Leiden, Zuid-Holland, Netherlands; Leiden University Medical Center & Healthplus.ai, Leiden, Zuid-Holland, Netherlands; Healthplus.ai, Amsterdam, Noord-Holland, Netherlands; Leiden University Medical Center, Leiden, Zuid-Holland, Netherlands; Leiden University Medical Center, Leiden, Zuid-Holland, Netherlands

## Abstract

**Background:**

C-reactive protein (CRP) is a nonspecific marker of inflammation and due to surgery alone CRP levels are increased post-operatively. Although multiple studies investigated the potential of post-operative CRP levels to predict post-operative infection, its discriminative capacity remained unclear. We aimed to describe the kinetics of CRP for post-operative patients with- and without a post-operative infection for different types of surgery in the first seven post-operative days.

**Methods:**

We conducted a single center cohort study in adult patients undergoing surgery between 2011 and 2021 with the use of whole digital case file extraction (big data). All patients that had either an infectious complication registered, non-prophylactic antibiotics prescribed and/or an infection related surgical re-intervention within 7 days after having undergone surgery, were labelled as having an infection. We analyzed the CRP kinetics in the first seven post-operative days for all these patients.

**Results:**

A total of 39.985 individual patients were included of which 12.002 patients had at least one CRP measurement and 4.014 patients had an infection in the first seven post-operative days. CRP was measured in 66% of the patients with an infection and in 26% of the patients without an infection. In all patients CRP increased during the first two post-operative days regardless of infection status (Figure 1). In cardiothoracic, orthopedic, ear-nose-throat, general and urological surgery CRP decreased after day 2 or 3 in all patients but the absolute CRP value remained higher in the following days in patients with an infection compared to patients without an infection. In gynecological and oral surgery CRP decreased on day 3 in patients without an infection but not before day 4 in patients with an infection. CRP was not discriminative in neurosurgical or plastic surgery patients (Figure 2).

CRP kinetics in the first seven post-operative days for patients with- and without an infection

**Figure:**
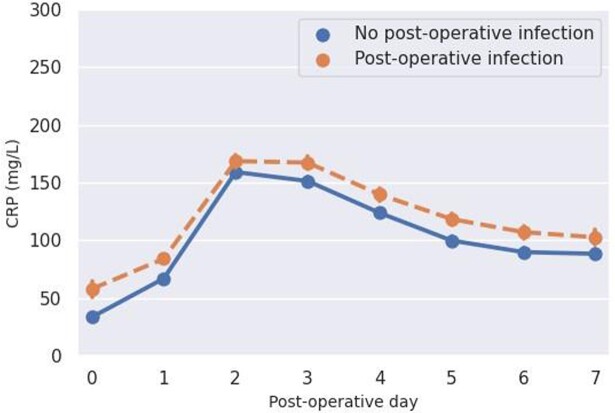

CRP kinetics for different subspecialties in the first seven post-operative days

**Figure:**
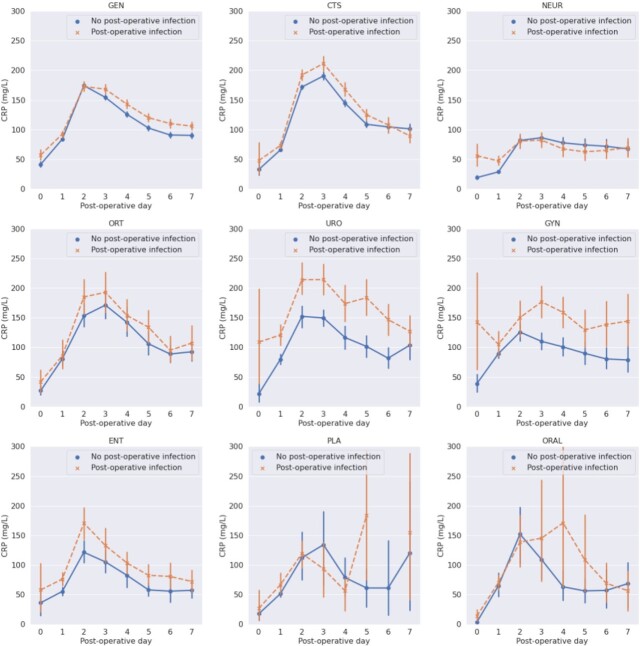

CTS = cardiothoracic surgery; ENT = ear-nose-throat surgery; GEN = general surgery; GYN = gynecological surgery; NEUR = neurosurgery; ORAL = oral surgery; ORT = orthopedic surgery; PLA = plastic surgery; URO = urological surgery.

**Conclusion:**

In the first 2 post-operative days CRP increases in both patients with- and without an infection which is probably due to the surgical procedure itself. After the second post-operative day differences in CRP kinetics develop between patients with- and without an infection, but how they differ depends, among other factors, on the type of surgery.

**Disclosures:**

**Siri L. van der meijden, MSc**, Healthplus.ai: Employee **Bart F. Geerts, MD. PhD.**, Healthplus.ai: Board Member.

